# Mapping length of inpatient treatment duration and year‐wise relapse rates in eating disordered populations in a well‐defined Western‐European healthcare region across 1998–2020

**DOI:** 10.1002/mpr.1960

**Published:** 2023-01-30

**Authors:** Peter Andersson, Esmail Jamshidi, Carl‐Johan Ekman, Kristina Tedroff, Jonnie Björkander, Magnus Sjögren, Johan Lundberg, Jussi Jokinen, Adrian E. Desai Boström

**Affiliations:** ^1^ Department of Clinical Neuroscience/Psychology Karolinska Institute Stockholm Sweden; ^2^ Centre for Clinical Research Dalarna Uppsala University Falun Sweden; ^3^ Stockholm Health Care Services Region Stockholm Stockholm Sweden; ^4^ Department of Clinical Sciences/Psychiatry Umeå University Umeå Sweden; ^5^ Centre for Psychiatry Research, Department of Clinical Neuroscience Karolinska Institutet, & Stockholm Health Care Services, Region Stockholm, Karolinska University Hospital Stockholm Sweden; ^6^ Department of Women's and Children's Health/Neuropediatrics Karolinska Institutet Stockholm Sweden

**Keywords:** ecological evidence, inpatients, severe anorexia nervosa, treatment guidelines, weight restoration

## Abstract

**Objectives:**

Updated international guideline recommendations for AN inpatient care rely on expert opinions/observational evidence and promote extended inpatient stays, warranting investigation using higher‐level ecological evidence.

**Methods:**

The study was conducted according to Guidelines for Accurate and Transparent Health Estimates Reporting (GATHER). Data encompassing 13,885 ED inpatients (5336 adolescents and 8549 adults) was retrieved from Swedish public health registries. Variables analyzed included (1) ED inpatient care opportunities, (2) unique number of ED inpatients and (3) mean length of ED‐related inpatient stays in age groups 15–19 and 20–88+, across 1998–2020.

**Results:**

Mean length of inpatient stays was inversely correlated to relapse to ED‐related inpatient care within the same year (*p* < 0.001, R‐squared_adj_ = 0.5216 and *p* < 0.00001, R‐squared_adj_ = 0.5090, in the 15–19 and 20–88+ age groups, respectively), independent of number of ED inpatients treated within a year in both age groups. Extending mean adolescent inpatient duration from 35 to 45 days was associated with a ∼30% reduction in the year‐wise relapse rate.

**Conclusions:**

Mean length of ED‐related inpatient treatment stays was associated with reduced relapses to inpatient care within the same year, which could be interpreted as support for recommendations to include a stabilization phase in inpatient ED treatment.

## 1 INTRODUCTION

Anorexia nervosa (AN) is a severely debilitating psychiatric illness characterized by a distortion of body perception, restrictive food consumption and fear of weight gain (American Psychiatric Association, 2013). AN has an estimated lifetime prevalence of 0.80% and a peak incidence at 13–18 years of age (Campbell & Peebles, 2014; Kappou et al., 2021; Udo & Grilo, 2018). Females are significantly overrepresented among those afflicted (Udo & Grilo, 2018). Inpatient treatment is commonly required in severe cases, with outcomes suggested to have improved overtime (Haas et al., 2021; Lindblad et al., 2006). However, the first year after discharge from an inpatient episode is associated with high risk of relapse (Berends et al., 2018) and treatment objectives are not achieved in a substantial proportion of cases admitted (Herpertz‐Dahlmann et al., 2014a; Wales et al., 2016). In a large 1‐year follow‐up study of specialized inpatient care in Germany, improvements in body weight and eating disorder symptoms were noted—effects that were maintained over time based on self‐reports of BMI at end of follow‐up. However, substantial heterogeneity in treatment response and high risk of relapse within a year of discharge (similar to previous findings (Berends et al., 2018)) were also observed, indicating the existence of subgroups prone to symptomatic deterioration post discharge. Longer illness duration, age and previous experience of inpatient treatment predicted unsatisfactory outcomes (Meule et al., 2021).

Guidelines for managing severe AN in the inpatient context emphasize weight restoration (*MARSIPAN: Management of Really Sick Patients with Anorexia Nervosa 2nd Edition CR189*, 2014; Robinson & Jones, 2018). These efforts typically begin with attempts to reinstate nutritional consumption through oral intake and, in cases where this fail, proceed to nasogastric tube‐feeding (NG‐feeding), sometimes on a compulsory basis. A longitudinal study of AN‐inpatients demonstrated increased clinical stability in patients who achieved weight gain exceeding 0.8 kg per week, validating the emphasis given to weight restoration. Yet, clinical deterioration over time still afflicted 29% of those that achieved the desired weight gain, indicating that current treatment regimens fail to elicit favorable responses in a substantial proportion of those treated (Lund et al., 2009). Non‐adherence to NG‐feeding is also common, reported to occur in nearly 30% of cases treated (Kells & Kelly‐Weeder, 2016). Furthermore, it has been argued that several characteristics of NG‐feeding are detrimental to prospects of long‐term recovery (Hart et al., 2013). In a comprehensive literature review, treatment drop out was reported to occur in 20%–51% of AN inpatients receiving care on a voluntary basis (32). Treatment drop‐out has been associated to shorter overall length of stay and lower body weight at discharge (Fassino et al., 2009).

Studies suggest that the role of inpatient care has changed over time. The recent widespread reservation of inpatient care for stabilization‐focused efforts in acute phases of AN has been suggested as contributing to recent increases in re‐admittance rates to inpatient care (Wiseman et al., 2001). In 2019, new evidence‐based guidelines recommended a reorientation of the role of inpatient care based on new observational evidence (level IV; B), that is, “*In order to reduce the probability of relapse, the final stage of inpatient therapy should aim to ensure that patients at least maintain their weight for a certain period and are prepared for the transition to an outpatient setting*” (Herpertz‐Dahlmann et al., 2014b). Longer inpatient treatment may, thus, be beneficial in facilitating weight stabilization maintenance. No previous study investigated the association between length of inpatient treatment stay and proportion of immediate relapses to inpatient care in relation to ED.

## 2 METHODS

This analysis was conducted in accordance with the Guidelines for Accurate and Transparent Health Estimates Reporting (GATHER) (Stevens et al., 2016). Using ecological data, the proportion of yearly relapses to ED‐related inpatient care and the mean length of inpatient treatment stays across both sexes, separated by age‐groups (i.e., 15–19 and 20–88+, respectively), were estimated. The underlying data encompass 13,885 unique ED inpatients (5336 adolescents and 8549 adults)—representing all ED inpatient care opportunities reported to the Swedish National Board of Health and Welfare in 1998–2020. Data was retrieved from an openly‐accessible registry reported by Swedish National Registers (openly‐available in Swedish: https://sdb.socialstyrelsen.se/if_dor/val.aspx; https://sdb.socialstyrelsen.se/if_lak/val.aspx). Data was available for the years 1998–2020 and were reported in accordance with ICD‐10 registered diagnoses at an aggregated level—thus, in the case of eating disorders, reflecting all reported inpatient care opportunities where F50—Eating Disorders was recorded as the primary diagnosis for the inpatient treatment period. Data on specific diagnostic categories associated with F50 were not available. Thus, the relative contribution of F50.0 Anorexia Nervosa to the studied F50 category, was not discernable. The following variables were available and retrieved on a year‐wise national basis for the years 1998–2020—separately for the two age‐groups: (1) ED inpatient care opportunities (primary diagnosis according to ICD‐10 being F50—Eating Disorders), (2) unique number of ED subjects receiving inpatient treatment (primary diagnosis according to ICD‐10 being F50—Eating Disorders) and (3) the mean length of ED‐related inpatient treatment stays (days, calculated by dividing the total length of stay across all included subjects by the total number of non‐unique ED‐related inpatient care opportunities). Additional variables that were available but excluded from the analysis included: regional stratification, total length of treatment stay (sum of days), total length of treatment stay per 100,000 inhabitants (sum of days per 100,000 inhabitants), unique number of ED subjects receiving inpatient treatment per 100,000 inhabitants. As the study was performed on a national level—regional level data was not considered relevant. Data measured per 100,000 inhabitants was also excluded. The exclusion of such data was motivated by the input variable consisting of mean length of ED‐related inpatient stays (days)—calculated by dividing the total days of treatment provided in inpatient care across all samples with the total number of ED inpatient care opportunities—which would provide identical values when conferred from the comparison of population‐adjusted values for mean length of stay and number of care opportunities. To enable replication of our results, extracted data is provided as Supplementary Material. As the study pertained to anonymized openly accessible data and did not involve recruitment of subjects, formal ethical approval from relevant authorities was not considered applicable. No samples were excluded from the analysis.

Initial analysis steps were performed in using Microsoft Excel for Microsoft 365 MO (Version 2204 Build 16.0.15128.20278) and included the following operations: (1) calculation of the year‐wise proportion of ED inpatients relapsing in the same calendar year (i.e., (*total number of care opportunities—total number of unique patients)/(total number of care opportunities*)). As a second step, (2) the interrelationship between variables of interest were illustrated in a combined chart type consisting of a clustered column (total number of patients) and two lines with markers (i.e., proportion of relapsing patients and average length of inpatient treatment stays—the latter with values presented in a secondary *Y*‐axis).

The association between year‐wise mean length of ED inpatient treatment (days) and the proportion of relapsing patients in the same year was evaluated in using R statistics software, version 4.2.0. The association analyses were performed separately for the two included age‐groups. First, the variables of interest were tested for normality. In the adult age‐group, the variable measuring proportion of ED relapses to inpatient care for each respective year was not normally distributed (*W* = 0.905, *p* = 0.0327) and, hence, subjected to transformation by Blom's method (Soloman & Sawilowsky, 2009) for the subsequent analyses (but—for illustrative purposes—not for figures). All other were normally distributed by Shapiro‐Wilk's tests (*p* > 0.05). Second, the variables were tested for an association with the total number of patients in inpatient care by Pearson correlations—as any such correlations could be expected to bias univariate association analyses between the two primary variables of interest. Neither variable correlated with the year‐wise total number of patients in ED inpatient care by Pearson correlations in the adolescent group (*p* > 0.1). By contrast, in the adult age group, both variables correlated with total number of inpatients (average length of inpatient treatment (days) [*r* = −0.55, *p* < 0.01]; Blom‐transformed proportion of ED relapses to inpatient care for each respective year [*r* = 0.66, *p* < 0.001]) (data not shown). Moreover, to investigate any general time trends, all variables were separately investigated for an association with year of measurement (i.e., 2008–2020) by univariate robust linear regression models (details of which specified below) in each age group separately. Third, the association between mean length of ED inpatient treatment (days) and the year‐wise proportion of relapsing patients in the same year was evaluated by robust linear regression models using the R–package “robustbase” (Todorov & Filzmoser, 2010), specifying recommended setting (KS2014), standard MM‐regression estimators (guaranteeing an acceptable compromise between high breakdown (i.e., 50%) and very high efficiency (i.e., 95%) (Yohai, 1987)). Subsequently—and to further reduce probabilities of bias from unaccounted for variations in the total year‐wise number of ED inpatients—robust multiple linear regression models were performed, contrasting these two variables, and adjusting for the total year‐wise number of ED inpatients. Lastly, to investigate whether the unique number of yearly ED inpatients acts as a mediator between length of inpatient stays and relapse rates in the 20–88+ age group, the above analysis was reperformed—adding an interaction term to account for any collinearity between these variables. *p*‐values <0.05 were considered significant. For retrieval of the source code used in the analysis, please contact the corresponding author.

## 3 RESULTS

**FIGURE 1:**
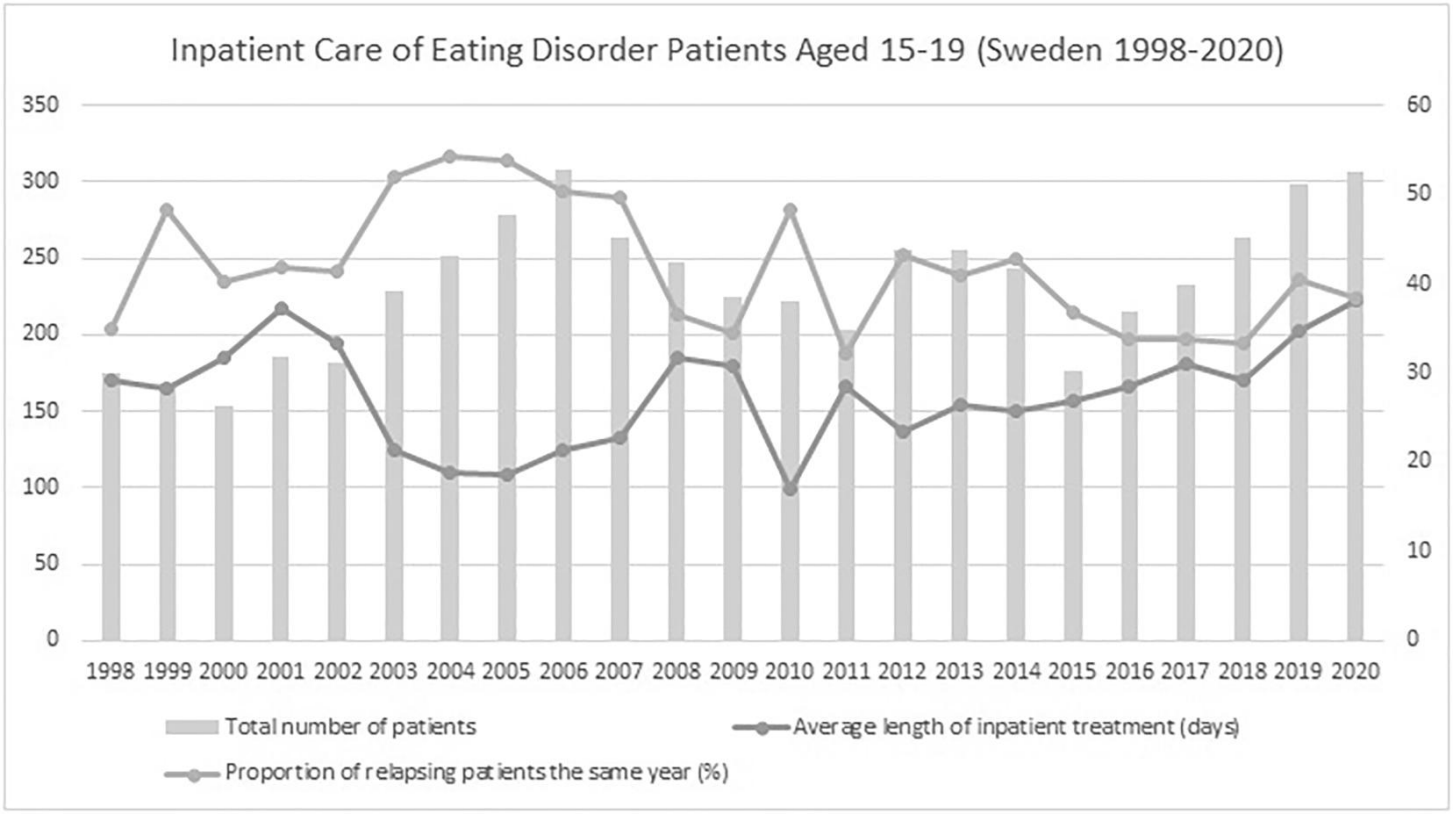
Total number of patients, average length of stay and proportion of relapses to inpatient care for ED patients aged 15–19 (Sweden, 1998–2020). Openly available data from the Swedish National Board of Health and Welfare was retrieved to illustrate development of key variables in relation to inpatient care for eating‐disorder patients aged 15–19 in Sweden (1998–2020). The left *y*‐axis depicts the total number of patients undergoing inpatient treatment for that respective year. The right *y*‐axis reflects the average length of inpatient treatment (days) and the proportion of relapsing patients for each respective year. The proportion of relapsing patients was, for each respective year, calculated by subtracting total care opportunities from total unique patients receiving inpatient care, and dividing the result with total care opportunities. Across 1998–2020 (1) the average total number of ED inpatients was 232 (SD = 44.8), (2) the average length of inpatient treatment was 27.6 days (SD = 5.8 days), (3) the proportion of patients relapsing in the same year averaged 41.8% (SD = 7.0%). Since 2015, the total number of ED inpatients exhibited a consecutive yearly increase totaling 73.8%. Visual inspection implicated an inverse relationship between average length of inpatient treatment (days) and proportion of relapsing patients in the same year, effects that appear especially pronounced in the periods 2002–2008, 2010 and 2012–2016.

**FIGURE 2:**
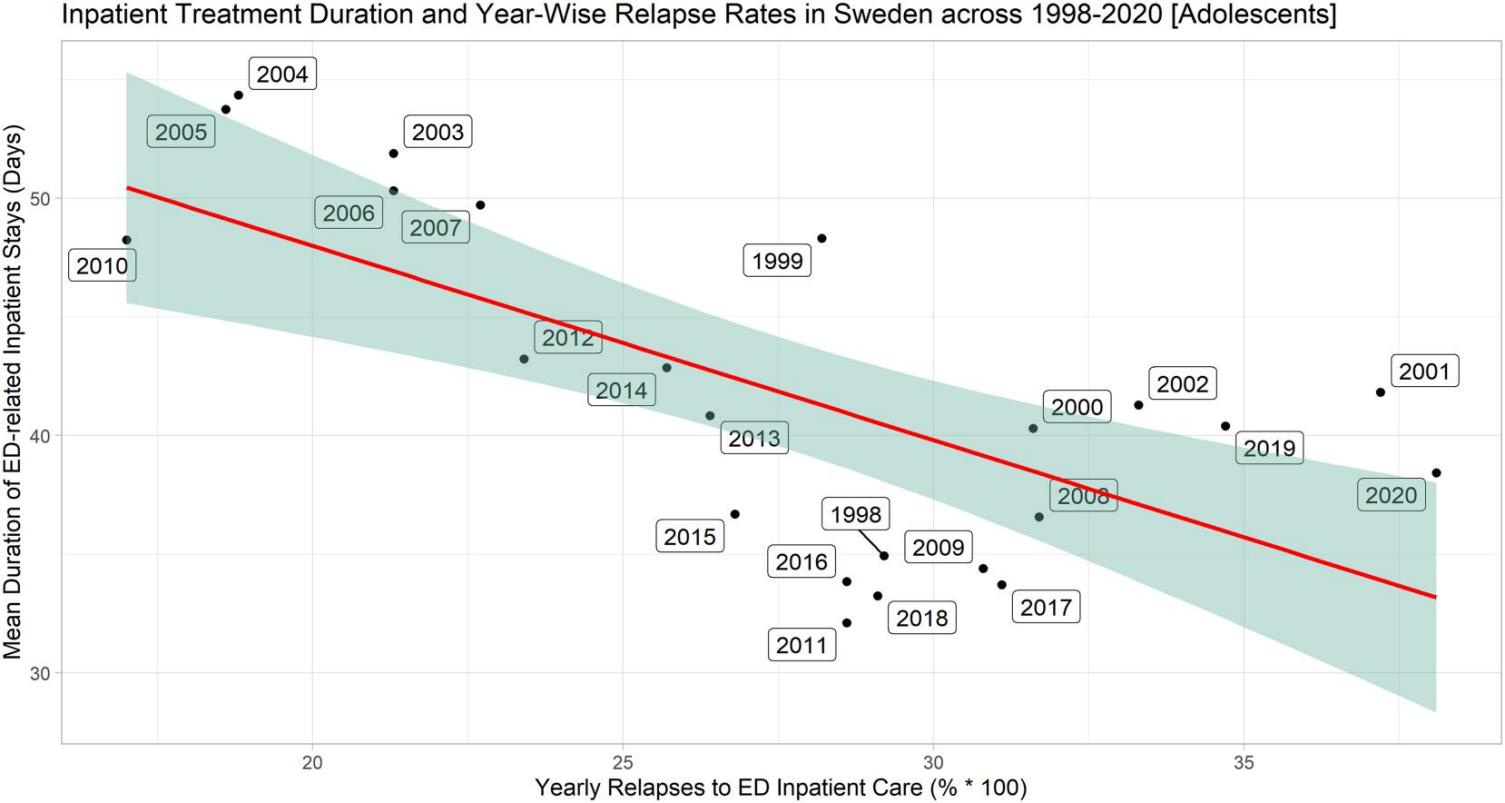
Correlation plot contrasting average length of inpatient treatment with proportion of relapses to inpatient care for ED patients aged 15–19 (Sweden, 1998–2020). Openly available data from the Swedish National Board of Health and Welfare was retrieved to illustrate associations between the average length of inpatient treatment (days) with the proportion of ED relapses to inpatient care in the 15–19 age group for each respective year. Both variables were normally distributed by Shapiro‐Wilk's tests (*p* > 0.05) and neither correlated with the total number of patients in inpatient care (*p* > 0.1). Univariate robust linear regression models show that yearly mean length of inpatient stays is inversely correlated to risk of relapse to ED‐related inpatient care within the same year (coef. = −0.52, *p* < 0.001, R‐squared_adj_ = 0.5216)—indicating that extending mean adolescent inpatient duration from 35 to 45 days was associated with a ∼30% reduction in the year‐wise relapse rate to ED inpatient treatment (Figure 1).

**FIGURE 3:**
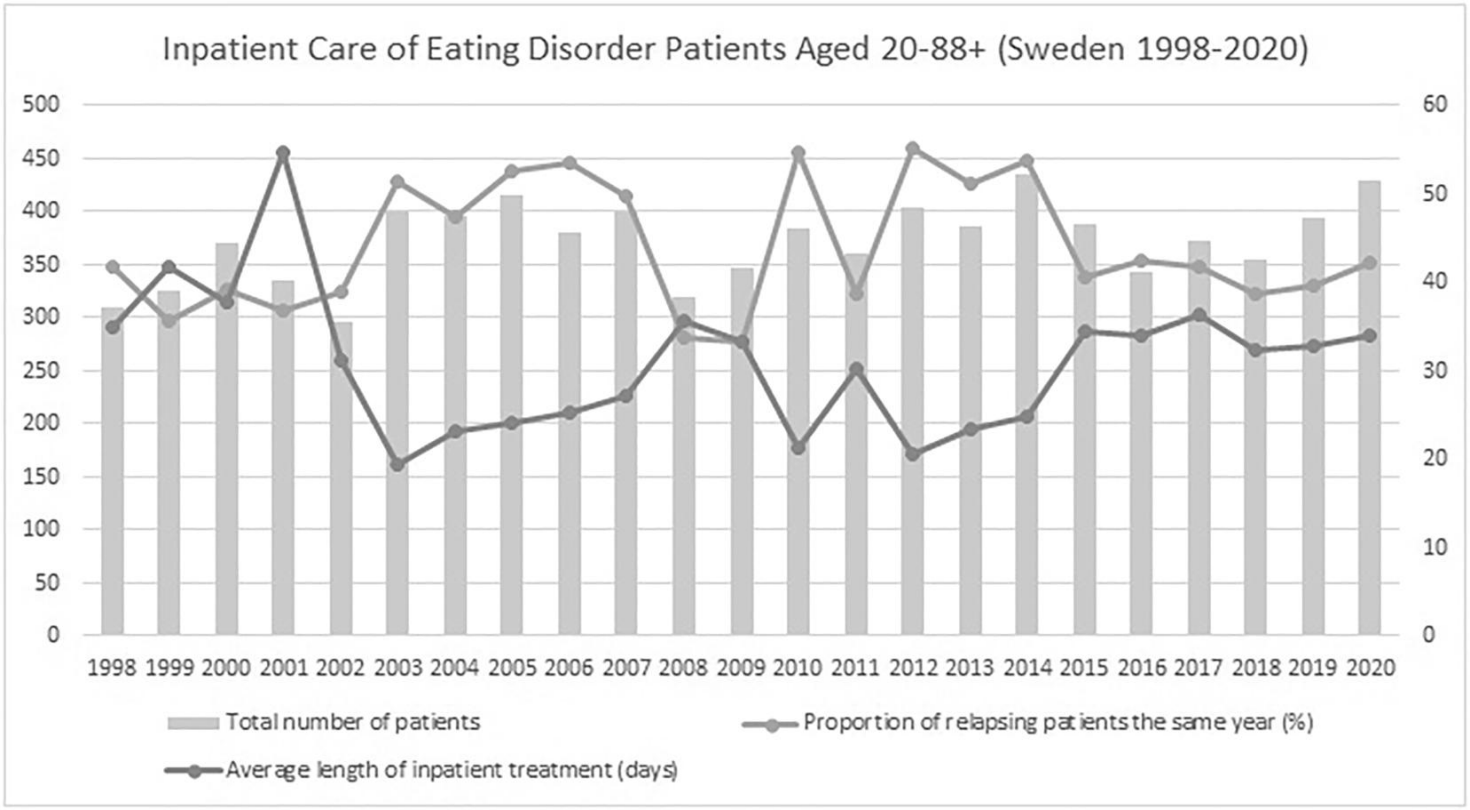
Total number of patients, average length of stay and proportion of relapses to inpatient care for ED patients aged 20–88+ (Sweden, 1998–2020). Openly available data from the Swedish National Board of Health and Welfare was retrieved to illustrate development of key variables in relation to inpatient care for eating‐disorder patients aged 20 and above in Sweden (1998–2020). The left *y*‐axis depicts the total number of patients undergoing inpatient treatment for that respective year. The right *y*‐axis reflects the average length of inpatient treatment (days) and the proportion of relapsing patients for each respective year. The proportion of relapsing patients was, for each respective year, calculated by subtracting total care opportunities from total unique patients receiving inpatient care, and dividing the result with total care opportunities. Across 1998–2020 (1) the average total number of ED inpatients was 372 (SD = 37.7), (2) the average length of inpatient treatment was 31 days (SD = 8.0 days), (3) the proportion of patients relapsing in the same year averaged 44.0% (SD = 7.2%). In contrast to data regarding inpatient treatment for individuals aged 19 and below, the total number of inpatients increased less drastically between the years 2015–2020. A consecutive yearly increase only took place during the last three years of that period (2018–2020) and the total increase between 2015 and 2020 totaled 10.8% – in contrast with corresponding increase of 73.8% in ED inpatients aged 15–19 for the same period (see Figure 1). Similarly, to the ED inpatient population aged 15–19, visual inspection implicated an inverse relationship between average length of inpatient treatment (days) and proportion of relapsing patients in the same year, effects that appear especially pronounced in the periods 2003–2008, 2010 and 2012–2015.

**FIGURE 4:**
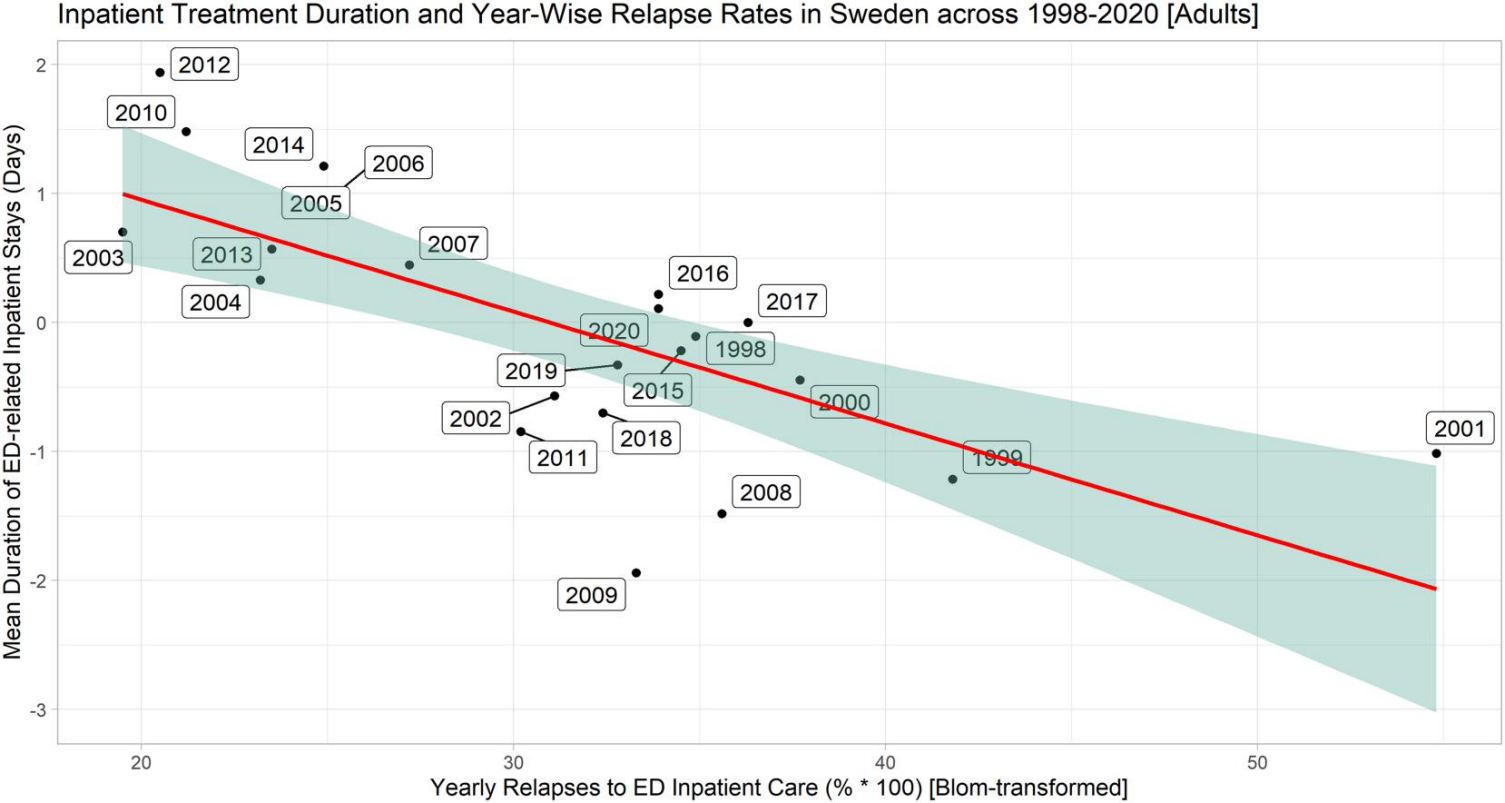
Correlation plot contrasting average length of inpatient treatment with normalized proportion of relapses to inpatient care for ED patients aged 20–88+ (Sweden, 1998–2020). Openly available data from the Swedish National Board of Health and Welfare was retrieved to illustrate associations between the average length of inpatient treatment (days) with the proportion of ED relapses to inpatient care for each respective year, in ED patients aged 20 and above. In contrast with ED inpatients aged 15–19, both length of inpatient treatment (days) and the blom‐normalized proportions of ED relapses to inpatient care were correlated to the total number of patients in inpatient care (*r* = −0.55, *p* < 0.01 and *r* = 0.66, *p* < 0.001; respectively) (**data not shown**). Univariate robust linear regression models show that length of inpatient treatment was inversely correlated to blom‐transformed proportion of relapses to inpatient care (coef. = −5.23, *p* < 0.00001, R‐squared_adj_ = 0.5090) (Figure 3), an association that was independent of the total number of ED subjects in inpatient care (Table 2).

In the adolescent group across 1998–2020, (1) the average total number of ED inpatients was 232 (SD = 44.8), (2) the average length of inpatient treatment was 27.6 days (SD = 5.8 days), (3) the proportion of patients relapsing in the same calendar year averaged 41.8% (SD = 7.0%). Since 2015, the total number of ED inpatients exhibited a consecutive yearly increase totaling 73.8% (Figure 1). Across 2008–2020, the unique number of ED inpatients exhibited a positive general time trend (coef. = 2.51, *p* = 0.037, R‐squared_adj_ = 0.1441) as measured by univariate robust linear regression models (**data not shown**). No such time trends were evinced in the case of relapse rates (*p* > 0.1) or average length of inpatient treatment (*p* > 0.1). We were able to confirm that length of inpatient treatment exhibited a moderate to strong correlation to the proportion of relapses to inpatient care in the same year for ED patients (coef. = −0.52, *p* < 0.0005, R‐squared_adj_ = 0.5216)—that is,—indicating that extending mean inpatient duration from 35 to 45 days was associated with a ∼30% reduction in the year‐wise relapse rate to ED inpatient treatment (Figure 2). Results were independent of the yearly total number of ED patients in inpatient care (Table 1). In the adult group and across 1998–2020, we were able to confirm similar findings, that is, length of inpatient treatment was inversely correlated to blom‐transformed proportion of relapses to inpatient care (coef. = −4.74, *p* < 0.005, R‐squared_adj_ = 0.5090), an association that was independent of the total number of ED subjects in inpatient care (Figures 3 and 4, Table 2). A complementary post‐hoc analysis in the adult age group—involving Blom‐transformation (Ludwig, 1961) of all variables to avoid violation of model assumptions ‐ demonstrated that length of inpatient treatment was inversely associated to yearly relapse rates (coef. = −0.62, *p* < 0.01, R‐squared_adj_ = 0.3907), independent of both the unique yearly number of ED inpatients and an interaction term encompassing the latter variable and year‐wise relapse rates (data not shown).

**TABLE 1:** Robust multiple linear regression models contrasting average length of ED inpatient treatment (days) to proportion of relapses to inpatient care (% * 100) and adjusting for total Nr. of ED inpatients aged 15–19 (Sweden, 1998–2020).

Parameter	Coef.	Std. Error	*t*‐Value	*p*
Intercept	51.38	5.95	8.63	3.50E‐08*
Proport. Relapses	−0.48	0.13	−3.86	9.64E‐04*
Total Nr. of ED inpatients	−0.02	0.02	−0.9	0.39

*Note*: Openly available data from the Swedish National Board of Health and Welfare was retrieved to study associations in the ages 15–19 between the average length of inpatient treatment (days) with the proportion of ED relapses to inpatient care for each respective year. Both variables were normally distributed by Shapiro‐Wilk's tests (*p* > 0.05) and neither correlated with the total number of patients in inpatient care (*p* > 0.1) (**data not shown**). Robust multiple linear regression models were performed contrasting average length of ED inpatient treatment (days) to proportion of relapses to inpatient care (% * 100), adjusting for the total number of patients in inpatient care. Adjusted R‐squared was 0.4905. *p*‐values <0.05 were considered significant (*).

Abbreviations: Coef, coefficient; Std. Error, standard error; *p*, *p*‐value; Proport. Relapses, proportion of ED relapses to inpatient care for each respective year (variable expressed in percentage * 100); Total nr. of ED inpatients, total number of patients in inpatient care.

**TABLE 2:** Robust multiple linear regression models contrasting average length of ED inpatient treatment (days) to proportion of relapses to inpatient care (% * 100) and adjusting for total Nr. of ED inpatients aged 20–88+ (Sweden, 1998–2020).

Parameter	Coef.	Std. Error	*t*‐Value	*p*
Intercept	38.10	12.98	2.93	0.00824*
Proport. Relapses	−4.74	1.36	−3.49	0.00231*
Total Nr. of ED inpatients	−0.02	0.04	−0.59	0.56

*Note*: Openly available data from the Swedish National Board of Health and Welfare was retrieved to study associations in the ages 20–88+ between the average length of inpatient treatment (days) with the blom‐transformed proportion of ED relapses to inpatient care for each respective year. In contrast to the previous analysis in the age range 15–19, both variables correlated with total number of inpatients (average length of inpatient treatment (days): *r* = −0.55, *p* < 0.01); blom‐transformed proportion of ED relapses to inpatient care for each respective year (*r* = 0.66, *p* < 0.001) (**data not shown**). Robust multiple linear regression models were performed contrasting average length of ED inpatient treatment (days) to proportion of relapses to inpatient care (% * 100), adjusting for the total number of patients in inpatient care. Adjusted R‐squared was 0.509. *p*‐values <0.05 were considered significant (*).

Abbreviations: Coef, coefficient; Std. Error, standard error; *p*, *p*‐value; Proport. Relapses, proportion of ED relapses to inpatient care for each respective year (variable expressed in percentage * 100); Total nr. of ED inpatients, total number of patients in inpatient care.

## 4 DISCUSSION

Recent updates to clinical guidelines for inpatient AN treatment recommend extending inpatient care to include a stabilizing period. However, this updated recommendation is based on lower‐quality evidence conferred from expert opinions and observational data. In a comprehensive analysis of 13,885 unique ED inpatient care opportunities (5336 adolescents and 8549 adults) performed in a large Western healthcare region across 1998–2020, we demonstrate that the mean length of ED inpatient treatment stays is inversely associated to risk of relapse to inpatient care in the same year. These associations were demonstrated in both adolescent and adult populations—and were independent of yearly variations in the total number of ED inpatients. Thus, we provide higher‐quality ecological real‐world evidence, indicating that ED inpatients could benefit from extended stays. The association between duration of care episodes and relapses were not affected by adjustments for total number of patients treated, which could be cautiously interpreted as suggesting that the overall care burden of services for ED patients was not the factor driving the association. Length of ED‐related inpatient treatment stays was inversely associated to the proportion of relapses to inpatient treatment in the same year and may in part reflect effects from higher BMI at discharge, extended facilitation of more comprehensive co‐ordination and planning for subsequent services by outpatient caregivers, better‐quality mental health care, or other potential unaccounted for factors.

There are some noteworthy limitations to the results presented herein. The observational nature of the research precludes inferences regarding causality. For example, the present study did not allow for directly investigating whether the association between increased length of stay and decreased risk of relapse could have been affected by specific confound. If decreased length of stay is associated with risk for more severe illness, those who stay in the hospital longer could have an intrinsically better prognosis. While it has not been possible to completely exclude major confound from any such effects, a recent systematic review and meta‐analysis by Kan et al.—evincing no associations between clinical features and length of admissions for AN ‐ contribute to reducing such concerns (Kan et al., 2021). Nevertheless, further studies accounting for illness severity at admission would be of value to confirm the results presented. It should also be noted that conducting controlled studies on the effects of treatment duration on outcomes in acute AN inpatients arguably raises several ethical concerns. In the absence of such data, results from observational research such as the work presented herein could provide heuristic value. Importantly, data availability issues also limited our ability to delineate the clinical group of interest, as the data available concerned all ED patients, whereas guidelines concerning extended inpatient care episodes concern AN specifically. However, in a recently published review of inpatient care for adolescent ED populations ‐ synthesizing results from 66 articles ‐ a majority of the included works reported results from populations where at least 50% were treated for AN (Isserlin et al., 2020), arguably suggesting that this group compose a significant proportion of the overall ED group in inpatient care, at least in adolescent populations. The present study pertained only to inpatient treatment episodes whereby an ED‐related diagnosis was labeled as the primary diagnosis for the inpatient treatment episode. This would contribute to reducing any confound from other reasons for inpatient admission, such as, for example, suicidality, self‐harm risk, strong compulsions, or harmful compensation method. Nevertheless, detailed extraction of recorded AN inpatient treatment stays would have been of value. However, such studies would be of value but was beyond the scope of this exploratory study. Furthermore, a potential confound that was impossible to control for was seasonal variation (longer length of stay is a potential source of confound on relapse potential for the same year when, for example, taking place in late December). Such effects would, however, be expected to affect each year equally. Thus, while potentially contributing to underreporting of relapse‐rates, it would be unlikely that the inability to account for relapses in the subsequent calendar year would have biased our main results on the association between length of inpatient treatment stays and yearly relapse rates. Regardless, accounting for relapse rates also during the subsequent calendar year would have been of value to provide a more comprehensive overview of any such associations. However, given the nature of the data, such analysis was not possible. Variables not satisfying assumptions for normality (i.e., relapse rates) were subjected to Blom‐transformation (Ludwig, 1961) in the adult age group. Thus, we advise caution in the interpretation of the regression estimate for this variable as direct comparison with other non‐Blom‐transformed variable estimates included in the same model may be misleading. The adolescent study group, however, included no such variable transformation—allowing for the comparison of regression estimates across included variables. Lastly, more detailed data could have allowed for more in‐depth investigation of the importance of factors such as weight stability pre‐discharge and the role of subsequent outpatient care in prevention of re‐admission, as well as relationships between treatment duration and long‐term outcomes. Carefully designed prospective studies seems warranted to address these knowledge gaps.

## AUTHOR CONTRIBUTIONS

Adrian E. Desai Boström conceived of the study and led the project. Adrian E. Desai Boström, Peter Andersson and Esmail Jamshidi wrote the first manuscript. Adrian E. Desai Boström performed the analysis of Swedish National Registry Data. All authors contributed to the evidence synthesis and contributed important input for revisions of study design and of the first draft of the paper. All authors contributed to the interpretation of data and analysis of results.

## CONFLICT OF INTEREST STATEMENT

The authors have no competing interests to declare.

## Supporting information

Supporting Information S1

## Data Availability

All data is openly available.
